# When Do Good Deeds Lead to Good Feelings? Eudaimonic Orientation Moderates the Happiness Benefits of Prosocial Behavior

**DOI:** 10.3390/ijerph17114053

**Published:** 2020-06-06

**Authors:** Weipeng Lai, Zhixu Yang, Yanhui Mao, Qionghan Zhang, Hezhi Chen, Jianhong Ma

**Affiliations:** 1 Department of Psychology and Behavioral Sciences, Zhejiang University, Hangzhou 310058, China; wplai@zju.edu.cn (W.L.); yangzhixu@zju.edu.cn (Z.Y.); yanhui.mao@cgu.edu (Y.M.); joannazhang@zju.edu.cn (Q.Z.); chenhezhi@aliyun.com (H.C.); 2 School of Economics and Management, Southwest Jiaotong University, Chengdu 610031, China

**Keywords:** happiness, prosocial behavior, eudaimonic orientation, hedonic orientation, person-activity congruence

## Abstract

Engaging in prosocial behavior is considered an effective way to increase happiness in a sustainable manner. However, there is insufficient knowledge about the conditions under which such a happiness effect occurs. From a person-activity congruence perspective, we proposed that an individual’s eudaimonic orientation moderates the effect of prosocial behavior on happiness, whereas hedonic orientation does not. For this purpose, 128 participants were assigned to play a game in which half of them were explained the benevolence impact of playing the game (the benevolence condition), and the other half played the same game without this knowledge (the control condition). Participants’ eudaimonic and hedonic orientations were assessed before the game, and their post-task happiness were measured after the game. The results showed that participants in the benevolence condition reported higher post-task positive affect than those in the control condition. Furthermore, this happiness effect was moderated by participants’ eudaimonic orientation—participants with high eudaimonic orientation reaped greater benefits from benevolence, and their hedonic orientation did not moderate the relationship between benevolence and happiness. The importance of the effect of person-activity congruence on happiness is discussed, along with the implications of these findings for sustainably pursuing happiness.

“The happiest person is the person doing good stuff for good reasons.”

*Kennon Sheldon*

## 1. Introduction

The happiness of humans is important for the sustainable development of society. Further, the sustainable development of society requires the contributions of people to the community as well as society [1]. Research has indicated that happy individuals care about the problems in society more than unhappy individuals [2]. Furthermore, happy people are more likely to donate money to charity and to volunteer [3]. Therefore, the pursuit of happiness has become a significant concern in various fields. Past studies have shown that directly pursuing happiness—such as by engaging in pleasurable and self-centered activities—does not result in happiness [4,5]. On the contrary, the eudaimonic activity model states that happiness can be achieved by engaging in eudaimonic activities [6]. The concept of eudaimonia originates from the writings of Aristotle and refers to the most virtuous, rational, and exemplary ways to live a good life [7]. Eudaimonic activities are those that are indicative of virtue, excellence, the best within individuals, and the complete development of their potentials [8]. Ryan and Martela [7] defined eudaimonia as “a good and valued way of living that can produce happiness, vitality, and wellness as its byproducts.” In other words, people feel good when they do good.

### 1.1. Prosocial Behavior and Happiness

A type of eudaimonic activity that draws considerable attention from researchers is prosocial behavior. Numerous studies have reported about the happiness benefits of prosocial behavior. For example, in one study, participants who were asked to help in a confederate search for a “lost” piece of paper reported more positive mood than those in the control group [9]. Similarly, in another study, participants who were given the opportunity to help someone reported more positive mood than those who were not allowed to help [10]. In another experiment, participants were made to play a simple computer game, and half of them were informed about the beneficence impact of playing the game. Compared to the control group, participants who were aware that they were performing a prosocial act experienced more positive affect and meaningfulness, as well as marginally more vitality [11]. Studies have also indicated that not only doing good deeds but also spending money on others can boost happiness. In one previous study, participants assigned to spend money on others reported higher levels of happiness than those who were asked to spend money on their own selves [12]. Similarly, another study indicated that people reported higher levels of happiness while recollecting a memory of spending money on others than those who recalled a memory of spending money on themselves [13].

The happiness obtained from prosocial behavior appears sustainable and is relatively resistant to adaptation [14]. In one experiment, participants were asked to spend money either on their own selves or on someone else for 5 days, and report their happiness levels at the end of each day. The results showed that the happiness levels of participants who spent money on themselves declined steadily over the 5-day period, whereas no such decline was observed among those who spent money on other people [14]. In a subsequent study, participants were made to play 10 rounds of an incentivized game in which they won some money in each round; participants could either keep the money for themselves or donate it to a charity. The participants reported their happiness after each round, which showed that the happiness of those who gave their winnings away declined more slowly than that of participants who kept their winnings [14].

Although prosocial behavior can increase and sustain happiness, such an effect is not inevitable. In an experimental study, participants were randomly assigned either to volunteer with elementary school-aged children 1–2 h per week for 10 weeks or to a wait-list control group. The results showed that participants who were assigned to volunteer demonstrated no change in their affective well-being compared to the control group [15]. This inconsistency makes it necessary for researchers to focus on the conditions under which the happiness benefits of prosocial behavior are most likely to emerge.

### 1.2. Person-Activity Congruence and Happiness

Although research on prosocial behavior has shown that it can boost happiness, less is known about the conditions that facilitate this boosting effect. Drawing on theoretical and empirical evidence, we propose that person-activity congruence is a critical determinant of the happiness boosting effect of prosocial behavior.

Person-activity congruence refers to the degree to which the pattern of an individual’s personality attributes matches the pattern of their behavior [16]. In other words, person-activity congruence means behaving in line with one’s true self.

According to the eudaimonic identity theory, living in accordance with one’s true self results in various happiness benefits, such as feelings of personal expressiveness and hedonic enjoyment [17]. Similarly, the self-concordance model states that people put more effort into achieving goals that fit their core values (i.e., self-concordant goals), and reap greater happiness benefits from attaining these goals [18,19]. Moreover, the self-determination theory states that intrinsically motivated activity—an activity that is performed based on personal values, interest, and enjoyment—is critical for achieving happiness [20]. Further, the person-activity fit is an important element in the positive-activity model of Lyubomirsky and Layous [21]. According to this model, person-activity fit, along with the features of positive activities and persons, moderates the effect of positive activities on well-being.

Recent empirical evidence has supported the idea that living in congruence with one’s true self results in greater happiness [18,22,23,24,25]. Individuals whose personal goals and life-story identities were consistent with their personality traits reported high levels of happiness [22]. Similarly, in another study, participants who attained their self-concordant goals reaped greater happiness benefits from the attainment [18]. Sheldon [23] assessed long-distance hikers’ motivation and happiness and found that completing long-distance hiking increased the happiness of only those who identified with the activity. Further, one study reported that spending money resulted in happiness only when the spending fit the participants’ personalities [24]. For example, introverted people are happier when they purchase books than while spending money at bars [24]. Another study showed that the positive relationship between prosocial spending and happiness was significant only among individuals with higher self-transcendence values (i.e., a concern for others and the community) [25].

To summarize, person-activity congruence is central to the achievement of greater happiness. However, studies have yet to examine the relationship between prosocial behavior and happiness from the person-activity congruence perspective. We thus propose that eudaimonic orientation can moderate the relationship between prosocial behavior and happiness.

### 1.3. Eudaimonic Orientation and Hedonic Orientation

Individuals differ in the ways they pursue happiness by initiating actions toward various life goals. Eudaimonic and hedonic orientations are two types of orientations toward happiness [26,27]. Eudaimonic orientation is defined as the extent to which an individual seeks meaning, authenticity, excellence, and growth, whereas hedonic orientation refers to the extent to which an individual seeks pleasure and comfort [27,28]. Eudaimonic and hedonic orientations can coexist as they are based on different ways of conceptualizing and pursuing a good life. Furthermore, both are beneficial for happiness. Eudaimonic orientation was found to be linked to feelings of meaningfulness, elevation, and self-connectedness; on the other hand, hedonic orientation is associated with feelings of carefreeness, positive affect, and low negative affect [28]. However, empirical evidence has indicated that eudaimonic orientation is associated with prosocial values and behavior, whereas hedonic orientation is not associated with prosociality [27,29]. Therefore, in terms of person-activity congruence, eudaimonic orientation is related to prosocial behavior whereas hedonic orientation is not.

From the perspective of person-activity congruence, we propose that those with high eudaimonic orientation will consider a prosocial goal as a self-concordant one and that congruently engaging in prosocial behavior will result in greater happiness benefits from prosocial behavior. In other words, eudaimonic orientation will moderate the positive relationship between prosocial behavior and happiness. We also propose that hedonic orientation will not moderate the relationship between prosocial behavior and happiness, as hedonic orientation is unrelated to prosociality.

### 1.4. The Current Study

This study aimed to examine the roles of eudaimonic and hedonic orientations in moderating the effect of prosocial behavior on happiness. For this purpose, we adopted the benevolence game paradigm used by Martela and Ryan [11], in which participants were invited to spend 15 min playing a computer game, and only half of them were informed about the benevolence impact of the gameplay. We then measured the participants’ post-task happiness and task performance. Additionally, participants’ trait happiness, eudaimonic orientation, and hedonic orientation were measured prior to the task. Data and analysis code are publicly available at https://osf.io/4r27t/.

From the person-activity congruence perspective, we propose the following two hypotheses:

**Hypothesis** **1.** *Eudaimonic orientation moderates the effect of benevolence on happiness; thus, those with high eudaimonic orientation will report greater post-task happines*

**Hypothesis** **2.** *Hedonic orientation does not moderate the effect of benevolence on happiness.*

## 2. Materials and Methods

### 2.1. Participants

A total of 131 students were recruited from Zhejiang University through the university’s online message board. Each participant was compensated with 15 RMB (about $2.1) for participation. Three participants were excluded as they did not pass the attention check. In this study, the attention check comprised two items in the questionnaire stating, “To check your attention, please select strongly disagree.” Failure to select this response in either of the two items was considered failing the attention check. The final sample comprised 128 participants, of which 92 (71.9%) were women, and the average age was 20.48 (SD=2.37). A sensitivity power analysis conducted with G*Power 3 suggested that with this sample size (N=128), we had 80% power to detect an effect of $f^{2}=0.062$, which was a small-to-medium sized effect. The study protocol was approved by the research ethics committee of the Department of Psychology and Behavioral Sciences at Zhejiang University, China, and informed consent was obtained from all participants.

### 2.2. Procedure

Participants were invited to the laboratory and were seated in front of a shielded computer. After reading and signing an informed consent form, the participants reported their trait happiness, eudaimonic orientation, and hedonic orientation. Thereafter, the participants were instructed to play a computer game for 15 min, following which they completed a second questionnaire for determining their post-task happiness. Finally, the participants completed a questionnaire on their demographic data.

### 2.3. Benevolence Game Paradigm

To determine the effect of benevolence, we adopted a paradigm that was previously used by Martela and Ryan [11]. We randomly assigned half the participants to the benevolence condition and the other half to the control condition. Participants in both conditions were invited to play a game from freerice.com for 15 min. Additionally, only participants in the benevolence condition were informed of the benevolent impact of playing the game.

The specific instructions for participants in the benevolence condition were as follows:

“This is a simple game in which you will be asked to select the correct answer of a multiplication problem from among four alternatives. For each answer that you get right, the sponsors will send money equivalent of 10 grains of rice to the United Nations World Food Programme, who will use the money to save and change lives.”

The instructions for the control condition were as follows:

“This is a simple game in which you will be asked to select the correct answer of a multiplication problem from among four alternatives. For each answer that you get right, you will earn ten points. Let’s see how many points you can get.”

Participants in the benevolence condition could see the logo of World Food Programme on their screens, whereas for the control condition, the website was altered to hide all information related to rice donation.

### 2.4. Measures

Measurements that did not already have Chinese-language versions were translated from English to Chinese and verified through a standard translation and back translation procedure [30].

#### 2.4.1. Eudaimonic and Hedonic Orientations

Eudaimonic and hedonic orientations were measured using the Hedonic and Eudaimonic Motives for Activities-Revised (HEMA-R) scale developed by Huta [27]. The HEMA-R comprises 5 items for assessing eudaimonic orientation (e.g., seeking to contribute to others or the surrounding world), and 5 items to assess hedonic orientation (e.g., seeking pleasure). Thereafter, the respondents were asked to report the degrees of both orientations with which they typically approach their activities on a seven-point scale ranging from 1 (not at all) to 7 (very much). The Cronbach’s alphas for eudaimonic and hedonic orientations in this study were 0.80 and 0.81, respectively.

#### 2.4.2. Trait Happiness

To assess participants’ trait happiness, we used the Subjective Happiness Scale (SHS) [31]. The SHS is a 4-item scale developed to assess an individual’s overall happiness. The Cronbach’s alpha for the SHS was 0.87 in the present study.

#### 2.4.3. Sense of Prosocial Impact

Participants’ sense of prosocial impact was used as a manipulation check. Sense of prosocial impact was assessed using the Beneficence Scale [11], which has four items (e.g., “The things I do contribute to the betterment of society.”; 1—not at all true to 7—very true; α = 0.88). The participants were instructed to “Think about how you felt during the gameplay” in rating these four items.

#### 2.4.4. Post-Task Happiness

Participants reported their current positive and negative affect on the Positive and Negative Affect Schedule (PANAS; 1—not at all to 7—extremely; $\alpha_{positive}$ = 0.93; $\alpha_{negative}$ = 0.76) [32]. Thereafter, they completed the Satisfaction with Life Scale (SWLS) [33], in which they indicated their agreement with 5 items on a 7-point scale ranging from 1 (strongly disagree) to 7 (strongly agree). However, the fifth item—“If I could live my life all over again, I will change almost nothing”—was found to be poorly correlated with the total score (r=0.44), and was therefore excluded from further analysis. The Cronbach’s alpha for the remaining 4 items was 0.81.

According to Diener’s tripartite model of subjective well-being [34], subjective well-being is the scientific term for happiness and it is an evaluation of a person’s life based primarily on three primary components: positive affect, negative affect, and life satisfaction [35]. Therefore, in this study, we used positive affect, negative affect, and satisfaction with life as three indices for participants’ post-task state happiness. However, past experimental studies have only reported a consistent increase in positive affect after prosocial behavior [11,36]. Therefore, we focused our analysis on positive affect. For transparency, we included the results on negative affect and satisfaction with life.

#### 2.4.5. Task Performance

For every correct answer the participants got right, they got ten points. Task performance was measured with the number of points the participants got in total during the gameplay.

#### 2.4.6. Demographic Information

Participant’s gender and age were collected as demographic information.

## 3. Results

### 3.1. Descriptive Analysis

The means, standard deviations, and zero-order correlations of the study variables are shown in Table 1. Age and gender were found to be not significantly correlated with any of the happiness variables; therefore, we excluded participants’ ages and genders from further analysis.

### 3.2. Happiness Benefits of Benevolence

A comparison between the benevolence condition and the control condition was presented in Table 2. First, our manipulation was successful; participants reported a greater sense of prosocial impact in the benevolence condition compared to the control condition (t(125)=7.84, p<0.001). Then, we checked whether the randomization of participants’ assignments to the two groups was successful. As expected, there were no differences found in participants’ trait happiness, eudaimonic orientation, and hedonic orientation between the benevolence and control conditions. Thereafter, we assessed whether the participants in the two conditions differed in terms of post-task positive affect, negative affect, and satisfaction with life. The two-sample *t*-test revealed a significant difference in positive affect between the two groups. Participants in the benevolence condition reported higher positive affect than those in the control condition (t(126)=2.68, p=0.008). Moreover, the results remained the same when trait happiness was added as a covariate (t(125)=2.67, p=0.009). However, participants in the two conditions did not differ in terms of negative affect and satisfaction with life. The results remained the same when trait happiness was added as a covariate (for negative affect *t*(125) = −0.36, *p* = 0.719; for satisfaction with life *t*(125) = −0.14, *p* = 0.892). As negative affect and satisfaction with life did not vary between the two conditions, we excluded them from further moderation analyses. In addition, the difference in task performance between the benevolence condition and the control condition was not significant (*t*(125) = −0.14, *p* = 0.890).

### 3.3. Moderation Analysis for Eudaimonic Orientation

We expected that individuals with high eudaimonic orientation would reap more positive affect benefits in the benevolence condition compared to those in the control condition. To test this, we conducted a linear regression with the benevolence and control conditions, eudaimonic orientation, and an interaction term of benevolence and eudaimonic orientation predicting the post-task positive affect. The results are reported in Table 3.

As shown in Table 3, the moderation effect of eudaimonic orientation was significant (b=0.18, 95% CI [0.00, 0.35], t(124)=1.99, p=0.049).

**Figure 1:**
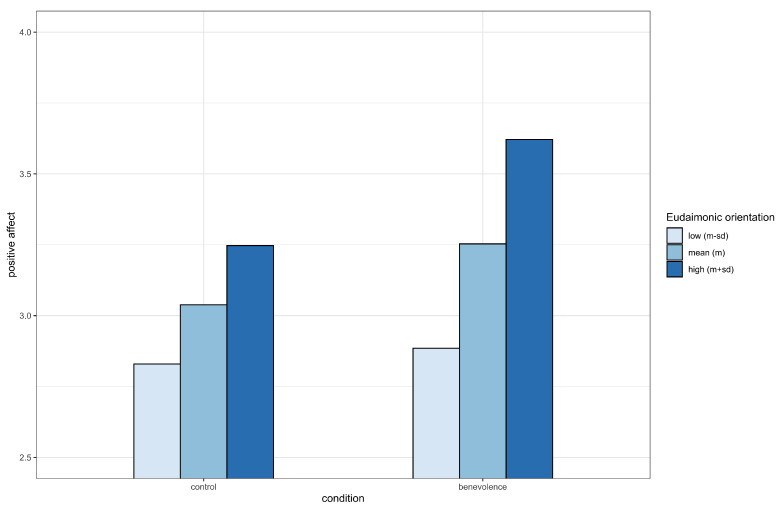
Eudaimonic orientation moderates the effect of benevolence on positive affect.

Further, simple slope analysis revealed that there was a significant positive affect benefit from benevolence for individuals with high (b=0.37, 95% CI [0.15, 0.60]) or average eudaimonic orientation (b=0.21, 95% CI [0.06, 0.37]). For participants with low eudaimonic orientation, there was a non-significant difference in positive affect between the benevolence and control conditions (b=0.06, 95% CI [−0.17, 0.28]) (see Figure 1).

### 3.4. Moderation Analysis for Hedonic Orientation

We expected that hedonic orientation will not moderate the effect of prosocial behavior on positive affect. As shown in Table 4, the moderation effect of hedonic orientation was not significant (*b* = −0.11, 95% CI [−0.28, 0.07], *t*(124) = −1.22, *p* = 0.223).

## 4. Discussion

The present study aimed to examine the moderating roles of eudaimonic and hedonic orientations in the effects of prosocial behavior on happiness. First, we found higher levels of positive affect after the game in the benevolence condition as compared to the control condition. This result is consistent with previous findings that demonstrated the positive link between prosocial behavior and happiness [11,12,37]. One possible alternative explanation for the higher levels of positive affect in the benevolence condition than in the control condition is that participants in the benevolence condition may have a better task performance and therefore reported higher levels of positive affect as compared to participants in the control condition. Our results showed that the task performance did not differ between the two conditions, which ruled out this alternative explanation. Moreover, we found that the effect of benevolence on happiness was moderated by eudaimonic orientation, which supports hypothesis 1. In other words, the higher one’s eudaimonic orientation, the stronger the effect of benevolence on happiness. Further simple slope analysis revealed that this positive effect of prosocial behavior on happiness was only for those with moderate to high eudaimonic orientation. Furthermore, as predicted in hypothesis 2, hedonic orientation was found to not moderate the effect of benevolence on happiness.

### 4.1. Contributions and Implications

The present study contributes to the literature on happiness in several ways. Our findings highlight the importance of person-activity congruence in understanding the pathway to happiness. Although prior studies have suggested that the effect of prosocial behavior on happiness is a “functional universal” and detectable across lifespans and the world [38,39] and that benevolence may be a “basic wellness enhancer” [40], the current findings suggest that the effect of prosocial behavior on happiness depends on the extent of individuals’ person-activity congruence. These findings are consistent with those of Hill and Howell [25], who found that the relationship between prosocial spending and happiness was only significant for individuals with high self-transcendence values. Therefore, further research on happiness should focus on person-activity congruence rather than on the activities alone. Furthermore, the notion of person-activity congruence has implications for happiness interventions. When designing interventions for boosting happiness, the interventions should be customized for each individual based on their person-activity congruence.

The second contribution of this study is that it extends the literature on the happiness benefits of prosocial behavior by examining the moderating role of eudaimonic orientation. Researchers have proposed that there is a potential path to sustainable happiness through a positive feedback loop between prosocial behavior and happiness, in which engaging in prosocial behavior results in increased happiness, which in turn encourages people to engage in further prosocial behavior [41,42,43]. If this is the case, the present study’s findings indicate that eudaimonic orientation may act as a catalyst in this positive feedback loop. People with high eudaimonic orientation reap greater happiness benefits from prosocial behavior, and thereby accelerate the upward spiral between prosocial behavior and happiness. On the contrary, people with low eudaimonic orientation will not feel happier after engaging in prosocial behavior and thus do not enter the positive feedback loop between prosocial behavior and happiness. This is consistent with the evidence that people with high eudaimonic orientation demonstrate high levels of happiness [28] and engage in prosocial behavior [29].

### 4.2. Limitations and Further Directions

The experiment presented in the current article is subject to a number of limitations. First, the nature of the relationship between eudaimonic orientation and the happiness benefit of benevolence in this study is correlational rather than causal. Therefore, further research is required to examine the causal role of eudaimonic orientation in the happiness boosting effect of prosocial behavior by experimentally manipulating participants’ eudaimonic orientation. Second, state happiness was not assessed prior to the game. Although we assessed participants’ trait happiness via the Subject Happiness Scale prior to the game and found that participants in the benevolence condition did not differ in trait happiness from those in the control condition, we did not measure state happiness prior to the game, which made it unclear whether there were any differences between benevolence condition and control condition in state happiness before engaging in the game. Moreover, the lack of measurement of state happiness prior to the game made it difficult to directly compare how state happiness has changed as a direct effect of the manipulation. Future studies should consider assessing state happiness twice (once before the task and once after task) using the same scale, which allows the test of state happiness differences between conditions prior to the task and more importantly the test of within-person differences in state happiness as a direct effect of the experimental task. Third, the benevolence game in the present experiment incurred no cost for the benefactor. Unlike the benevolence game, economic games in behavioral economics (e.g., the third-party punishment game and the public good game) often incur a cost to the participants’ prosocial choice. Future studies could use such an economic game to test whether prosocial behavior in costly forms has the same positive effect on happiness and whether this effect is also moderated by an individual’s eudaimonic orientation. Fourth, the moderating effect of hedonic orientation on the link between prosocial behavior and happiness was not significant in the current experiment. However, this might be due to insufficient statistical power to detect relatively small effects for the moderating influence of hedonic orientation. Thus, future research needs to replicate these results with larger samples. Fifth, the main outcome variable in the current study was a positive affect rather than specific emotions. However, prosocial behavior and person-activity congruence might have different effects on different emotions (e.g., joy, moral elevation, pride). Thus, future research needs to examine the effect of prosocial behavior and person-activity congruence on specific emotions. Another limitation is that we only examined the short-term happiness benefit of benevolence. Because long-term happiness can vary from short-term benefits [44], further research with a longitudinal design should be undertaken to investigate whether eudaimonic orientation can predict the long-term happiness benefits of prosocial behavior. A final limitation is the imbalance between the male and female sample sizes, with 71.8% female participants in our study. However, our results showed that gender did not significantly correlate with any other variables in this study (see Table 1). Therefore, the gender imbalance in our sample may not be a threat to our conclusion.

## 5. Conclusions

People feel good by doing good. However, it is not the good deed per se that leads to happiness, but the congruence between the good deed and the individual. Therefore, to become happier, an individual must live in accordance with his or her true self.

## Figures and Tables

**Table 1:** Means, standard deviations, and zero-order correlations of the study variables across conditions.

	*M*	SD	1	2	3	4	5	6	7	8
1. Positive affect	3.04	0.96	-							
2. Negative affect	1.24	0.33	0.14	-						
3. Satisfaction with life	3.90	1.11	0.09	0.03	-					
4. Benevolence condition	0.50	0.50	0.23 **	−0.03	−0.02	-				
5. Eudaimonic orientation	5.25	0.90	0.22 *	0.03	0.23 *	0.03	-			
6. Hedonic orientation	5.14	0.95	0.06	−0.02	0.15	−0.05	0.15	-		
7. Gender	0.72	0.45	0.16	−0.02	−0.10	0.00	−0.09	0.10	-	
8. Age	20.48	2.37	0.09	−0.15	−0.01	−0.06	0.09	0.11	−0.08	-
9. Trait happiness	4.69	1.25	−0.01	−0.11	0.60 ***	0.01	0.13	0.08	−0.07	−0.02

Note: Sample size *N* = 128. * *p* < 0.05; ** *p* < 0.01; *** *p* < 0.001.

**Table 2:** Comparison between the benevolence and control conditions

	Benevolence Condition		Control Condition		*t*-Test		Cohen’s *d* and 95% CI		
	*M*	SD	*M*	SD	*t*	*p*	*d*	Lower	Upper
Sense of prosocial impact	5.28	0.91	4.02	0.91	7.84	<0.001	1.39	1.00	1.77
Trait happiness	4.70	1.16	4.67	1.34	0.16	0.874	0.03	−0.32	0.37
Eudaimonic orientation	5.28	0.90	5.22	0.91	0.33	0.740	0.06	−0.29	0.41
Hedonic orientation	5.08	0.89	5.19	1.02	−0.61	0.543	−0.11	−0.46	0.24
Positive affect	3.26	0.98	2.82	0.88	2.68	0.008	0.47	0.12	0.82
Negative affect	1.23	0.26	1.25	0.39	−0.38	0.707	−0.07	−0.42	0.28
Satisfaction with life	3.88	1.15	3.92	1.07	−0.18	0.858	−0.03	−0.38	0.31
Task performance	1397.66	162.91	1401.88	181.75	−0.14	0.890	−0.02	−0.37	0.32

Note: Benevolence condition *N* = 64, Control condition *N* = 64.

**Table 3:** Regression results using eudaimonic orientation as a moderator.

	Positive Affect	
	(1)	(2)
Benevolence	0.22 **	0.21 **
	(0.08)	(0.08)
Eudaimonic orientation	0.23 *	0.23 *
	(0.09)	(0.09)
EUD * Benevolence		0.18 *
		(0.09)
Constant	3.04 ***	3.04 ***
	(0.08)	(0.08)
Observations	128	128
R${}^{2}$	0.10	0.13
Adjusted R${}^{2}$	0.09	0.11
F Statistic	7.00 ** (df = 2; 125)	6.10 *** (df = 3; 124)

Note: * *p* <0.05; ** *p* <0.01; *** *p* <0.001. values in parentheses indicate the standard errors of regression coefficient. EUD = Eudaimonic orientation.

**Table 4:** Regression results using hedonic orientation as a moderator.

	Positive Affect	
	(1)	(2)
Benevolence	0.22 **	0.22 **
	(0.08)	(0.08)
Hedonic orientation	0.07	0.06
	(0.09)	(0.09)
HED * Benevolence		−0.11
		(0.09)
Constant	3.04 ***	3.04 ***
	(0.08)	(0.08)
Observations	128	128
R${}^{2}$	0.06	0.07
Adjusted R${}^{2}$	0.04	0.05
F Statistic	3.92 * (df = 2; 125)	3.12 * (df = 3; 124)

Note: * *p* <0.05; ** *p* <0.01; *** *p*< 0.001. values in parentheses indicate the standard errors of regression coefficient. HED = Hedonic orientation.
